# Assessing the suitability of cell counting methods during different stages of a cell processing workflow using an ISO 20391-2 guided study design and analysis

**DOI:** 10.3389/fbioe.2023.1223227

**Published:** 2023-08-03

**Authors:** Colleen Richards, Sumona Sarkar, Jennifer Kandell, Richard Snyder, Uma Lakshmipathy

**Affiliations:** ^1^ Science and Technology Team, Pharma Services Group, Thermo Fisher Scientific, San Diego, CA, United States; ^2^ National Institute of Standards and Technology (NIST), Gaithersburg, MD, United States

**Keywords:** cell processing, biomanufacturing, cell count, viability, cell therapy, gene therapy, NIST, ISO

## Abstract

Cell counting is a fundamental measurement for determining viable cell numbers in biomanufacturing processes. The properties of different cell types and the range of intended uses for cell counts within a biomanufacturing process can lead to challenges in identifying suitable counting methods for each potential application. This is further amplified by user subjectivity in identifying the cells of interest and further identifying viable cells. Replacement of traditionally used manual counting methods with automated systems has alleviated some of these issues. However, a single cell type can exhibit different physical properties at various stages of cell processing which is further compounded by process impurities such as cell debris or magnetic beads. These factors make it challenging to develop a robust cell counting method that offers a high level of confidence in the results. Several initiatives from standards development organizations have attempted to address this critical need for standardization in cell counting. This study utilizes flow-based and image-based methods for the quantitative measurement of cell concentration and viability in the absence of a reference material, based on the tools and guidance provided by the International of Standards (ISO) and the US National Institute of Standards and Technology (NIST). Primary cells were examined at different stages of cell processing in a cell therapy workflow. Results from this study define a systematic approach that enables the identification of counting methods and parameters that are best suited for specific cell types and workflows to ensure accuracy and consistency. Cell counting is a foundational method used extensively along various steps of cell and gene therapy. The standard used in this study may be applied to other cell and gene therapy processes to enable accurate measurement of parameters required to guide critical decisions throughout the development and production process. Using a framework that confirms the suitability of the cell counting method used can minimize variability in the process and final product.

## 1 Introduction

Cell counting measurements are used in cell and gene therapy applications to evaluate cell viability and concentration to assess quality and quantity of cells for use in a variety of processes. In gene therapy workflows, viral production cells are transfected with plasmid DNA to generate viruses, including lentivirus (LV). These cells need to be carefully counted to ensure that the amounts of key components such as cell culture nutrients, transfection agent, and plasmid DNA are appropriate for the cell number present in the culture vessel. The viable cell density at the time of transfection can greatly impact the resulting vector production and impact the downstream process steps and product quality (Segura et al., 2007; Manceur et al., 2017). Cell counts should therefore be performed using a cell counting method that is reproducible and provides accurate results in order to maximize the yield of high-quality virus. The lentivirus produced can be designed to harbor genes encoding the chimeric antigen receptor (CAR) (Levine et al., 2017), which is then used to create CAR-T cells for cell therapy products.

The T cells required for generation of CAR-T therapies can be acquired by isolation from leukapheresis products or from peripheral blood mononuclear cells, and there are two main methods used to select these cells from the bulk population. Positive selection utilizes beads that bind to the cells of interest and allow the removal of non-target cells (Neurauter et al., 2007), while negative selection involves beads that bind to and remove non-target cells, leaving the desired cells. The beads used in positive selection may also activate the cells, while cells isolated by negative selection require separate activation steps (Brimnes et al., 2012; Noaks et al., 2021). One of the most important factors in both isolation and activation is the number of cells present in the sample, and the resulting bead to cell ratio. The ratio of beads to cells and the seeding density used post-isolation have a direct impact on cell expansion post-isolation (Neurauter et al., 2007; Ghaffari et al., 2021), which is essential for scale-up in cell therapy product development. When beads are attached to the cells, cell counting instruments using automated algorithms can be skewed by the presence of the beads, which makes achieving accurate cell counts especially challenging and influences the selection of an appropriate counting method.

Scaling cell expansion can be especially challenging for developing autologous cell therapy products, where it may not be possible to obtain a large number of healthy cells from a patient. Transitioning from autologous to allogeneic cells may help increase availability of cell therapy products by increasing the starting cell number and sourcing cells from healthy individuals (Depil et al., 2020), and clinical trials utilizing allogeneic products have shown success (Cruz et al., 2013; Brudno et al., 2016). Optimizing expansion is an essential part of the production of cell therapy products, and accurate cell counts play a vital role in scale-up and scale-out culture conditions (Scibona and Morbidelli, 2019). Evaluating the quality of a cell counting method begins with an experimental design that includes samples at varying dilutions, replicates to allow for statistical analysis, and clear criteria to determine suitability (“ISO 20391-1, 2018 Biotechnology–Cell counting–Part 1: General guidance on cell counting methods” 2018; Sarkar et al., 2017; Lin-Gibson et al., 2016). The experimental methods utilized in this study were designed in accordance with ISO 20391-2 (“ISO 20391-2, 2019: Biotechnology—Cell counting—Part 2: Experimental design and statistical analysis to quantify counting method performance” 2019; Huang et al., 2021). This standard offers guidance on sample preparation, required replicates and dilution levels, as well as evaluation of pipetting and/or user error and requires the reporting of several metrics including, for example, a proportionality index and %CV (coefficient of variation) across replicate observations (Sarkar et al., 2019).

**FIGURE 1:**
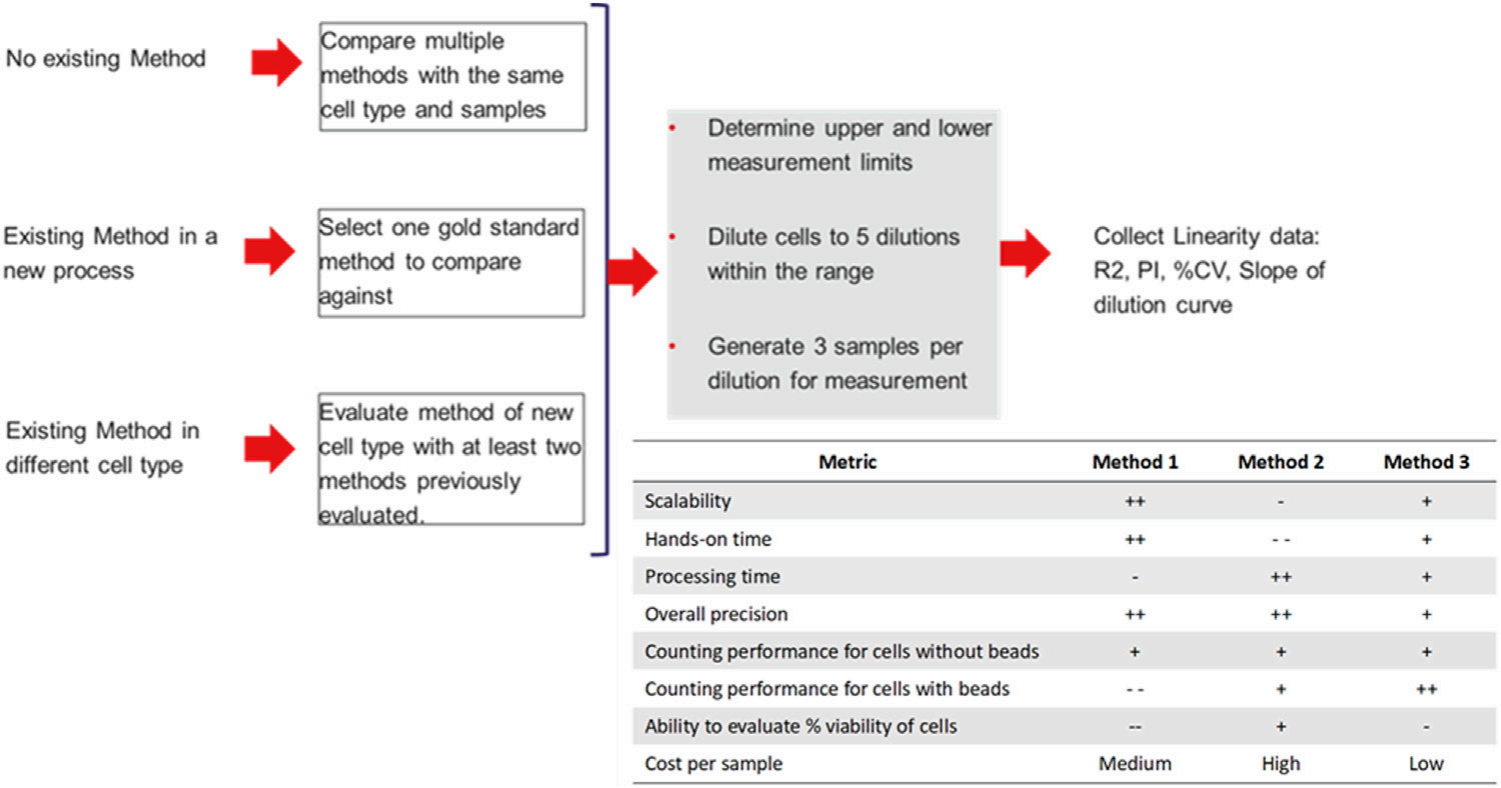
Proposed flowchart for evaluation of new methods and integration of new cell types into cell counting workflows. Additionally, results of the given evaluation comparing three different evaluation methods given with positives and negatives given to indicate suitability of each method in various areas, where higher “+” indicates a more suitable method, and higher “-” indicates a less suitable method. Relative cost is given per sample analyzed.

The guidance offered by the ISO 20391-2 standards was used to evaluate the performance of three counting methods and four sample types to determine the most appropriate method for measurement of cell count and viability throughout the example cell therapy production process. Additionally, a flowchart is shown in Figure 1 of an approach to evaluate new counting methods and integration of new cell types to aid researchers in qualifying new counting methods for use in non-GMP and GMP laboratory settings. This flowchart outlines recommended steps to be taken in integrating new cell processing steps and methods while being flexible to best fit into each laboratory’s individual processes. The flowchart also includes proposed evaluation criteria for use in determining the most suitable cell counting method, and the results of the given study in those categories.

## 2 Materials and methods

### 2.1 Sample preparation

A one-quarter cryopreserved Leukopak (Stem Cell Technologies, Cat #200-0132) was thawed using the PlasmaTherm Blood Program (Barkey) until no ice remained, then diluted 1:4 in wash buffer containing HBSS (Hanks’ Balanced Salt Solution, Gibco, Cat #14025092), ICSR (CTS™ Immune Cell SR, Gibco, Cat #A2596101), and EDTA (UltraPure™ 0.5M EDTA, Invitrogen, 15575020). Then, a sample was taken for cell counting method evaluation. Preliminary cell counts were performed to determine the estimated concentration in the leukopak and compare to the value given by the manufacturer. For leukopak cell counts, the blood sample was mixed with a lysis buffer to remove red blood cells and incubated. Cell counts were performed on Method 3 only using a method for whole blood samples.

Ficoll-Paque gradient separation was used to isolate peripheral blood mononuclear cells from the Leukopak. The thawed Leukopak was carefully layered onto Ficoll (Cytiva, Cat #17144002) in conical tubes (Thermo Cat #AM12501). These tubes were centrifuged to create the density gradient, and the buffy coat was removed and washed to generate a cell solution containing PBMCs for evaluation. From these cells, an approximate cell count was performed using Method 2 to evaluate approximate concentration before creation of the dilution series. After the approximate concentration was measured, the dilution series was created as in Section 2.2 and all three methods were evaluated.

T cells were isolated using either positive or negative selection. The positive selection method used magnetic beads (Dynabeads™ Human T-Expander CD3/CD28, Gibco, Cat #11141D) that bind to the T cells to be isolated and are used to separate these cells from the bulk cell population. In the negative selection method (Dynabeads™ Untouched™ Human T cells Kit, Invitrogen, Cat #11344D), antibodies directed towards CD14, CD16, CD19, CD36, CD56, CD123, and CD235a are added to the cell solution and incubated. Then, magnetic beads are added to bind to the antibodies and remove unwanted cells, leaving enriched T cells. Samples were taken from both the positively and negatively selected T cells for use in cell counting evaluations. Again, approximate cell counts were measured for the beaded and non-beaded cells using Method 2, and then the dilution series was created and all three methods were evaluated.

### 2.2 Experimental design

**FIGURE 2:**
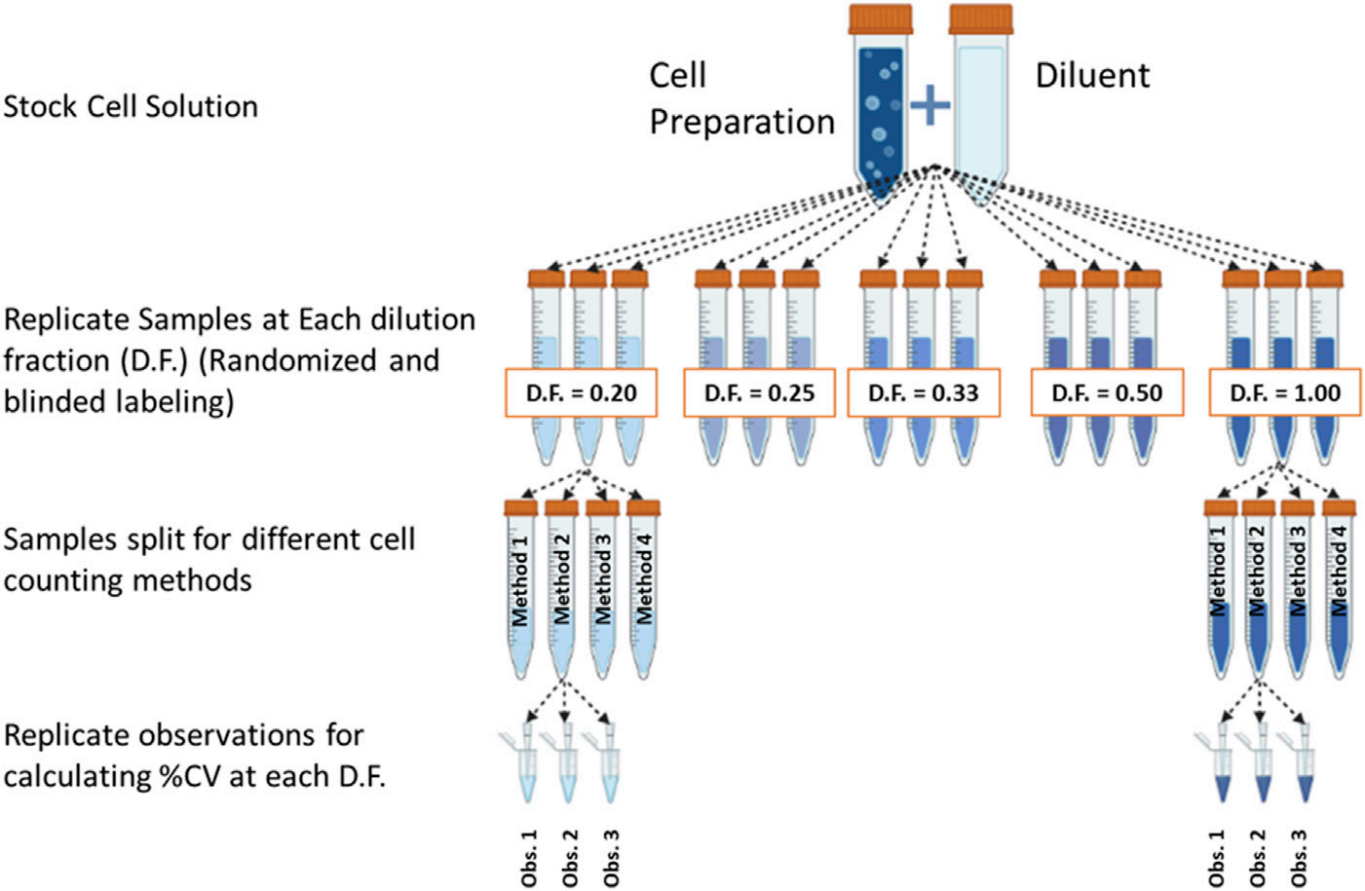
Dilution fraction design in accordance with recommendation from ISO Cell Counting Part 2. (Figure generated using BioRender).

The estimated cell counts for each sample type were used to generate a dilution series containing 5 dilutions (1:1, 1:2, 1:3, 1:4, 1:5) of the cell solution (Figure 2). The dilutions for all samples were created using isolation buffer composed of dPBS and Human Serum Albumin (DPBS, Gibco, Cat #14190144 and HSA, InVitroCare, Cat #2101). While these dilutions were useable for this experiment, it should be noted that an ideal dilution series more evenly spans the range from smallest to largest dilution. Concentrations for the dilution series ranged between 5X10^5 and 1X10^6 cells/mL to maintain all sample concentrations within the operating range of the instruments being evaluated. Evaluation of pipetting accuracy was not performed, due to the sensitive nature of the primary cells being evaluated. For each dilution, 3 sample tubes were generated for evaluation. Sample tubes within each sample type were assigned random ID numbers between 1 and 15 to prevent bias in counting measurements. Each tube was counted 3 times using each counting instrument and the same method as the initial count. The leukopak samples were counted using the instrument for Method 3 only, due to lack of protocols for whole blood on the other two instruments.

### 2.3 Counting methods

Each of the instruments used for cell counting were evaluated at each of the 5 dilutions. All methods utilize a different instrument and protocol, although all protocols used are intended for use in measurement of cell concentration and viability. Each sample was mixed well before cell counts were taken, and the sample volumes were confirmed to be sufficient to prevent air bubbles in automated counting systems.

For Method 1, samples were analyzed using parameters set specifically for PBMC and T cells. These parameters included cell size, circularity, brightness, and sharpness. This method utilized trypan blue and brightfield imaging to determine cell concentration and viability. The samples were loaded into the instrument, the cell type was selected, and the cell counts were performed. Method 2 utilized a cassette loaded with the stains necessary for cell counting, the cells were loaded into the cassette and the samples were analyzed using the cell count and viability measurement method. Both Method 2 and 3 utilized acridine orange (AO) and DAPI (4’,6-diamidino-2-phenylindole) to measure cell concentration and viability. There were two different analysis procedures used on Method 3 for evaluation. The first was used only for the samples taken directly from the Leukopak. This method used red blood cell lysis buffer that was added to the cells and incubated at 37°C for 10 min for staining. Then, samples were mixed and measured using the instrument’s analysis tool. The second procedure was used for the PBMC and T cell (both positive and negative selection) samples. For each sample the staining solution and the cells were mixed, and samples were loaded into the instrument for analysis using the cell count and viability measurement method. The default gating was found to be appropriate for the cell samples measured in this study using Method 2 and Method 3.

### 2.4 Data analysis

Values including total cell concentration, viable cell concentration, percent viability, and average cell diameter were recorded for each cell count measurement. Each of these values was tied to the specific sample number and replicate, and to the instrument on which the measurement was performed. Data analysis was performed using the COMET (Counting Method Evaluation Tool, https://github.com/usnistgov/COMET or https://cell-counting.shinyapps.io/COMET/) application as well as JMP data analysis software (JMP Statistical Discovery). Excel sheets used are provided in the supplementary data, and templates with information on how to use the software are available on the COMET website.

For each cell type the cell count data was added into the COMET template (available through the online tool) and uploaded into COMET for statistical analysis. The variance was assumed to be proportional to mean variance, the default order of smoothing polynomial was selected, the number of bootstrap iterations was set to 1,000, and the confidence level was set to 0.95. The proportionality index used was smoothed scaled sum squared error. Equations for calculation of proportionality index (PI) (Eq. 1) and smoothing (Eq. 2) are given below.

$${SSE}_{smooth,scaled}=\sum_{i}\sum_{j}{\left(\frac{e_{ij}^{\left(s\right)}}{{\hat{\lambda }}_{ij}}\right)}^{2}$$ (1)

$$Smoothing:y_{i}={\hat{\beta }}_{0}+{\hat{\beta }}_{1}DF_{i}+{\hat{\beta }}_{2}DF_{i}^{2}+{\hat{\beta }}_{3}DF_{i}^{3}+{\hat{\beta }}_{4}DF_{i}^{4}$$ (2)

COMET was used to calculate the %CV, R^2^, and PI values, using the equations and values given. JMP was then used to compare values for viability and cell concentration between instruments, using an ANOVA and pairwise comparison and alpha value of 0.05. After analysis, *p*-values were collected for each dilution of each cell type.

## 3 Results

### 3.1 Proportionality analysis

**FIGURE 3:**
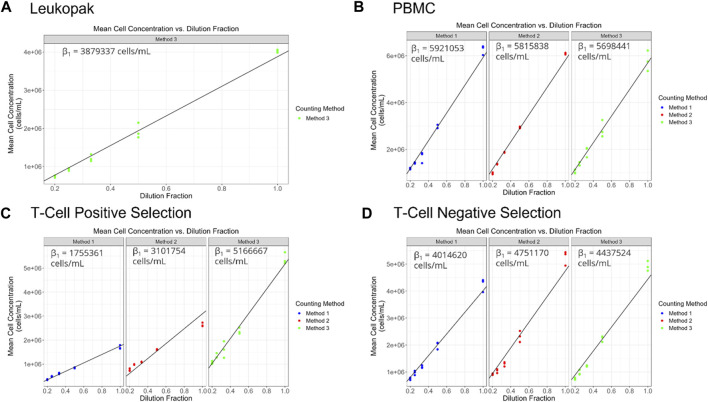
**(A–D)** display target dilution fraction on the *x*-axis and cell concentration (viable cell density in cells/mL) on the *y*-axis. Each point represents an average of the replicate observations for a particular replicate sample. The black diagonal line represents the fitted proportional model. The above button toggles prediction intervals computed using the fitted flexible (polynomial) model. Prediction intervals that do not overlap with the proportional model suggests potential non-linearity.

Viable cell density (cells/mL) was measured for the different cell preparations using different cell counting methods across a range of dilutions (Figure 2). The dilution series data was used to generate proportional model fits for each cell preparation and each cell counting method (Figure 3).

The equation for the proportional model fits is represented as,

$$\lambda_{ij}=\beta_{1}{DF}_{i}^{target}$$ (3)

Where i represents the index for the target dilution fraction ( ${DF}^{target}$ ), and j represents the sample index nested within the $i^{th}$ target dilution fraction. $\lambda_{ij}$ represents the true cell concentration for a particular $\left(i,j\right)$ and $\beta_{1}$ is the proportionality constant.

**FIGURE 4:**
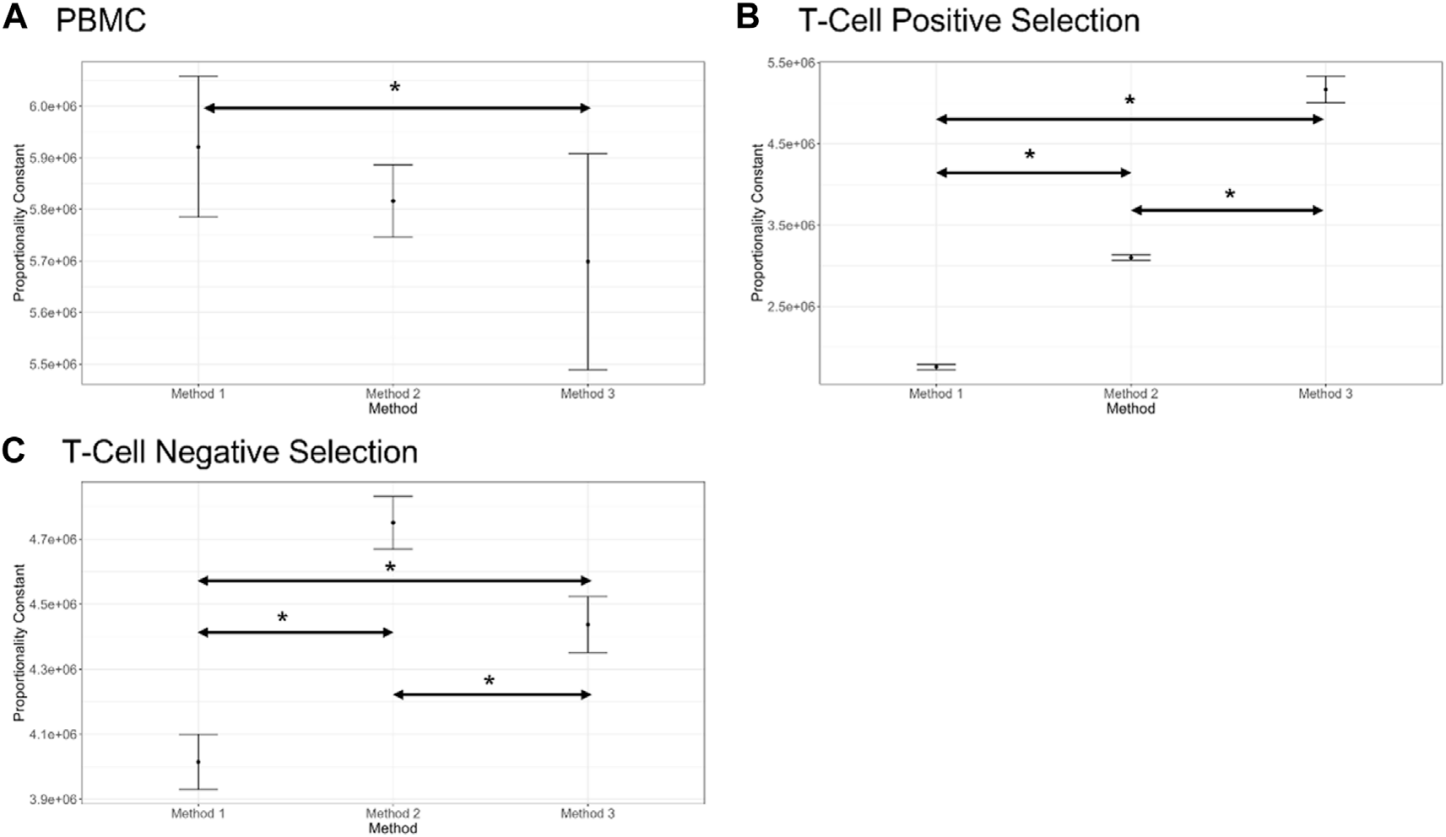
Proportionality constant (slope of the proportional model fit) estimates for each counting method and each cell preparation (shown in **(A–C)**), along with bootstrap confidence intervals (shown by bars on plots, 1,000 bootstrap iterations). Statistically different proportionality constants are indicated by an Asterix (*). Note that this difference does not suggest one method is more accurate than the other, only a difference in their proportional fits. Plots generated in COMET.

The proportionality constant, an estimate of the stock cell solution concentration is presented for each method and cell preparation in Figure 4 and can serve to identify systematic biases between methods. For PBMCs a significant bias was only observed between Method 1 and Method 3. For T cell positive selection and T cell negative selection cell preparations, all methods were significantly biased from one another. This indicates that, particularly for the T cell cell preparations, the different cell counting methods can be expected to provide different values for cell count. Therefore, in the absence of a reference cell counting method or cell count reference material, it is critical to understand the quality of these different measurement processes and the fitness-for-purpose of the methods when selecting a cell counting method. The dilution series data can also be evaluated with respect to linearity (i.e., allowing for a non-zero *y*-intercept) and comparing to an expected cell count value obtained by one of the cell counting measurement processes) (Supplementary Figure S1).

**FIGURE 5:**
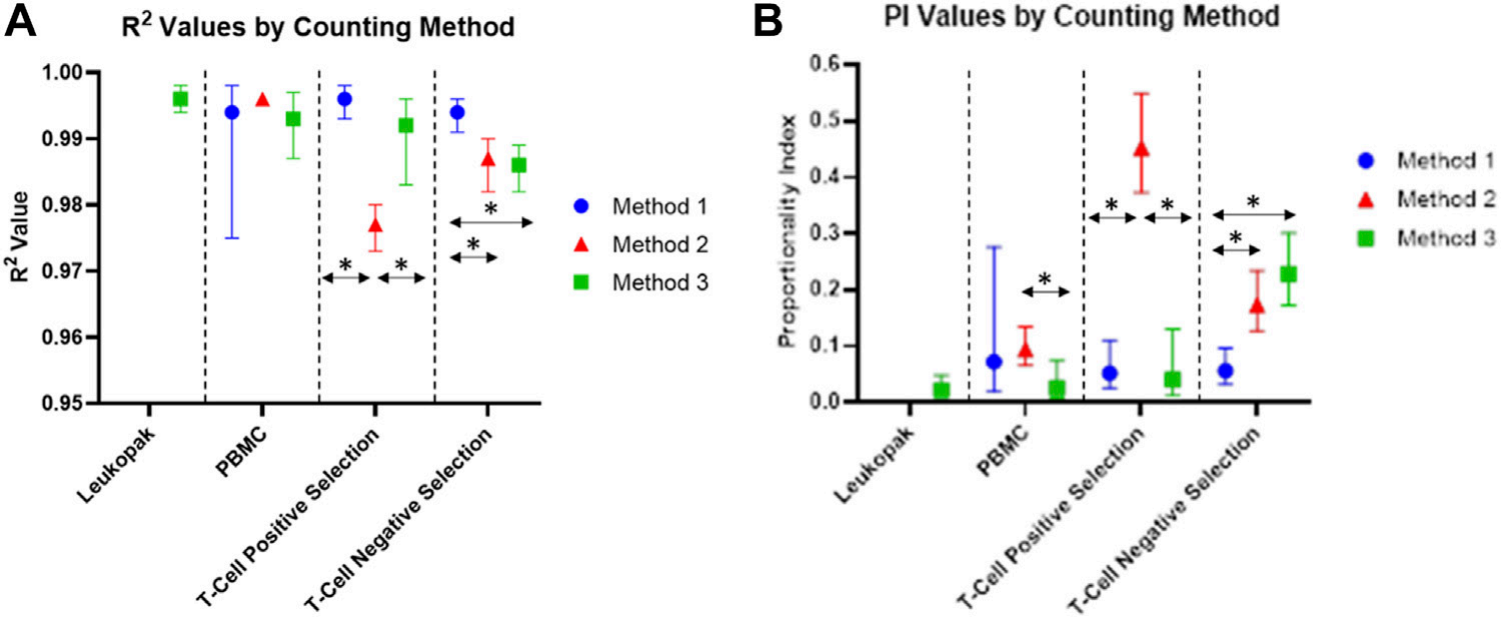
**(A)** R^2^ values of each proportional model fit for each counting method and cell preparation. **(B)** Proportionality Index (PI) values based on smoothed scaled sum of squared error from the proportional model fit to smoothed data. The vertical bars represent bootstrap confidence intervals computed at a confidence level of 95% and over 1,000 iterations.

The values for R^2^ for the proportional model fit for cells at each stage of the cell processing workflow are shown in Figure 5. R^2^ values remained above 0.9 for all samples, indicating that measured values for each dilution were close to the modeled values. Both random variability and systematic variability contribute to R^2^. There is no minimum value for R^2^ given by the standard used in this analysis, however values in the range generated are considered acceptable for this study. By looking further into the results and considering the proportionality index, differences emerge between the 3 methods evaluated here.

Proportionality index values for cells at each stage of processing are shown in Figure 5. The PI values indicate the level of deviation from proportionality for each measurement, after the effects of random variability have been reduced. The PI values for this experiment were calculated using the smoothed scaled sum squared error. For values calculated using this method, lower PI index values are representative of more proportional measurements, and higher PI values indicate greater deviation from proportionality. PI was lower for Method 3 for PBMCs and positively selected cells, compared to Method 1 and Method 2. For negatively selected cells, Method 3 had the highest PI of the three methods investigated. Method 2 had a significantly higher PI than Method 1 and Method 3 for positively selected cells, indicating a loss of proportionality in cell counting for these cells when Method 2 is used. The presence of beads in these samples may be contributing the loss in proportionality for Method 2, while Method 1 and Method 3 appear less affected by the presence of beads in the positively selected samples.

### 3.2 Precision of the cell counting methods

**FIGURE 6:**
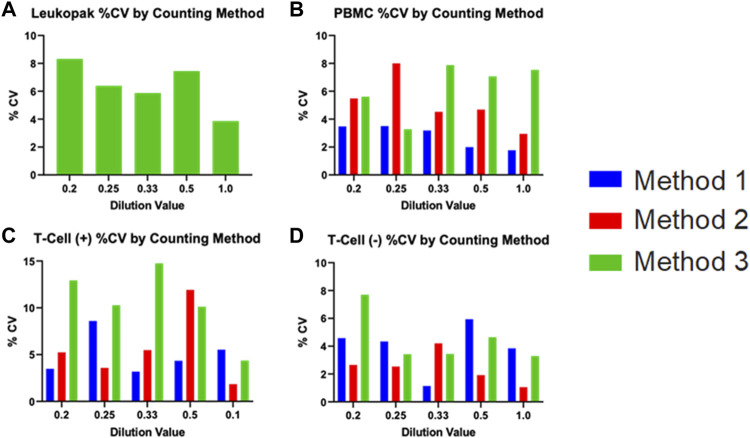
Coefficient of variation for each cell type measured, including **(A)** Leukopak, **(B)** PBMC, **(C)** T cell positive selection (T cells with beads attached), and **(D)** T cell negative selection (T cells without beads attached). Values are plotted separately for each dilution fraction. Only one instrument was evaluated for Leukopak samples. Each cell type has N = 15 per method.

The coefficient of variation across replicate observations from each sample was calculated for each instrument, with samples from each stage of the cell processing workflow analyzed separately. The %CV values indicate the precision of the instrument being analyzed. These values are displayed in Figure 6 For each cell sample. The %CV values for Leukopak, PBMC, and Negative Selection were <10% and values for Positive Selection were <15%.

### 3.3 Quality of the percent cell viability measurements

**FIGURE 7:**
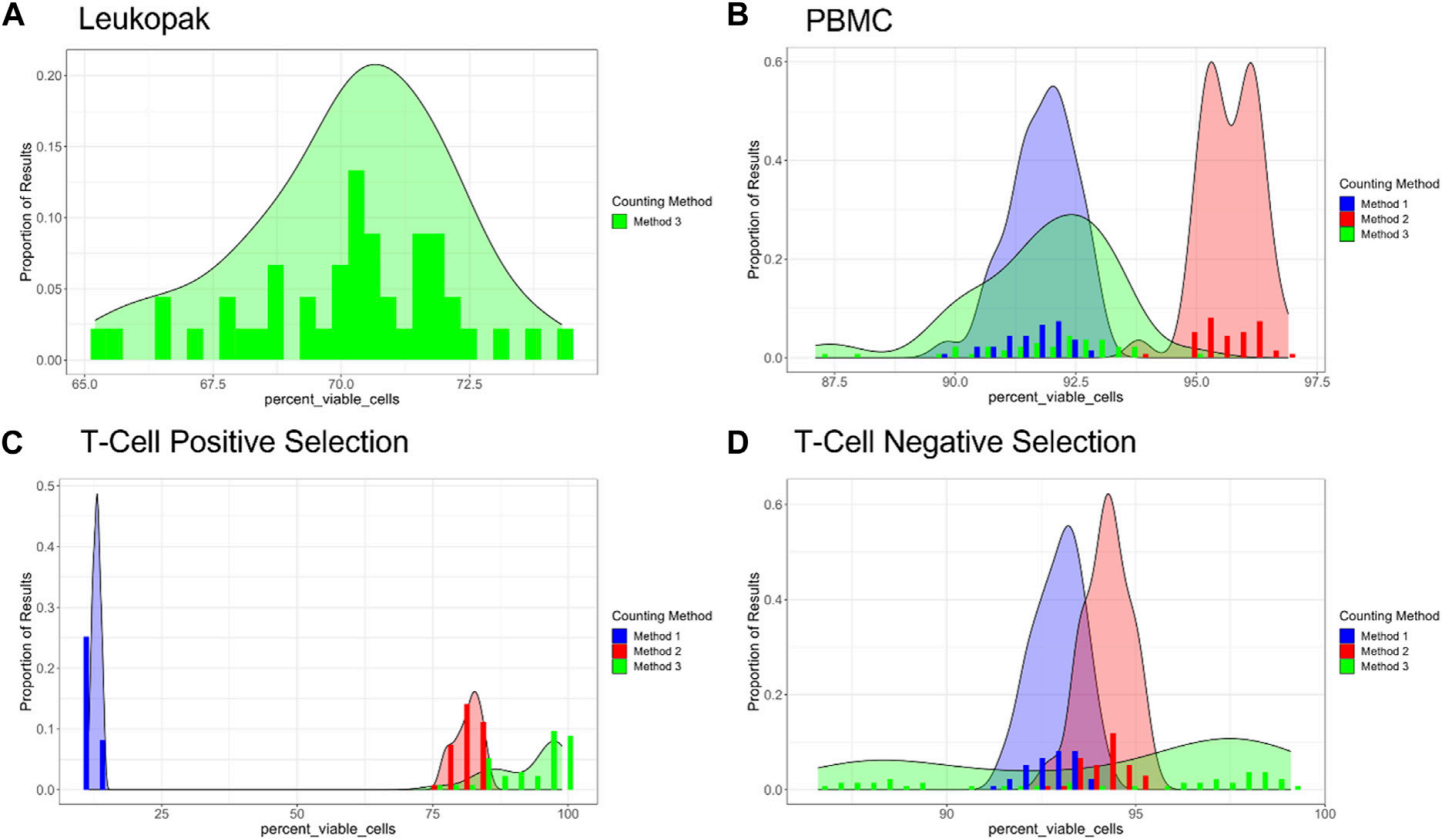
Percent viability for each cell type and method evaluated, where proportion of results is given on the *Y*-axis and percent viability is given on the *X*-axis. **(A)** Leukopaks, **(B)** PBMCs, **(C)** Positively selected T cells, and **(D)** Negatively selected T cells. Note that the *X*-axis ranges vary between plots. Plots generated by COMET.

COMET also provides analysis of the quality of cell viability measurements across the dilution series. In this analysis it is assumed that for an ideal % cell viability method, % cell viability would be the same for each sample regardless of dilution (in practical circumstances, it is expected that there may be some small amount of random variability in % viability across samples). In Figure 7, % cell viability is presented for the different methods and the different cell preparations, as a histogram to illustrate the frequency of % cell viability values across all of the samples evaluated in the dilution fraction study. For Leukopaks, % cell viability using method 1 ranges from approximately 65% to approximately 75% with a peak at approximately 71%. For PBMC samples, % cell viability peak frequencies occur at different values for % viability and have different but relatively narrow distributions in % cell viability across replicate samples, The difference in frequency distribution between Method 1 and Method 3 for the PBMC samples was not statistically significantly different (*p*-value = 0.97). For positively selected T cells, there is a large discrepancy between cell viability results from Method 1 compared to Methods 2 and 3. This may be due to interference from the beads in the positively selected T cell samples in Method 1, where the beads can be miscounted as non-viable cells, thus reducing the % viability reported by this method. For negatively selected T cells, the range of % cell viability values is between approximately 87% viability and 98% viability. Interestingly, Method 3 has a broader distribution in % cell viability and does not have a distinct highest frequency peak, as Methods 1 and 2 display. This indicates that Method 3 has higher variability in % cell viability than the other methods for negatively selected T cells. ANOVA analysis was also conducted to compare viable cell density and % cell viability across the different methods (Supplementary Table S1).

## 4 Discussion

### 4.1 Outcomes of evaluation at each stage of cell processing

Samples taken at different stages of a cell processing workflow present varying challenges based on the process impurities, phenotype and characteristics of the cells in each sample. This is especially evident in cell therapy production workflows, where cells often exist in heterogeneous populations or are bound to various types of beads. At the start of the workflow evaluated in this study, the leukopak samples contained a mix of various cell types including peripheral blood mononuclear cells, granulocytes, red blood cells, and platelets (Dean and Dean, 2005), which can potentially present challenges when using automated counting systems. However, the data produced in this study using Method 3 showed high linearity and the coefficient of variation was below 10%, indicating that the method used is acceptable for measuring whole blood samples. These results may be compared to methods like flow cytometry with the addition of counting beads to quantify cell density and identify specific cell types present in each sample.

When performing cell counts for PBMC samples, the cell counting parameters on Method 1 required user inputs to determine parameters used by the instrument’s measurement algorithm. Analysis of concentration and viability values indicated significant differences for viability between Method 1 and Methods 2 and 3, indicating that there may be potential for optimization of the pre-set parameters for PBMC samples on this instrument. COMET analysis showed goodness of fit (R^2^) over 0.98 for all instruments, while PI values remained low and showed significant differences only between Methods 2 and 3, in which samples were measured using 2 different formats (slide and cassette).

The final stage of cell processing evaluated in this study was post-isolation of T cells from the PBMC population. Negative selection did not significantly impact the cell counts recorded on any instrument. The slope of the dilution series was close to theoretical, linearity was high, and the coefficient of variation was low. The PI values were higher for negatively selected T cells than PBMCs, and there were significant differences between Methods 1 and 2 as well as between 1 and 3. This indicates that further studies should be performed to identify the source of these differences, however the results show that all methods are suitable for use on similar cell samples that contain a single cell type and do not include beads or other particles in the sample. The T cells which were positively selected and bound to magnetic beads presented issues with Method 1, which was likely due to the presence of dark beads in the sample identified incorrectly by the instrument as cells stained with trypan blue (Tennant, 1964; Strober, 2015). The viability measurements for this analysis were significantly different for samples measured on Method 1 compared to the other two methods. Concentration measurements were also impacted for Method 1 and Method 2. This is due to the presence of the beads interfering with quantification of viable cells for Method 1, and potentially due to the beads interfering with the microfluidic flow pattern utilized by the cassettes for Method 2. The interference of the beads also led to significantly increased PI values and decreased linearity for Method 2, when compared to the other two methods. While the viability was negatively impacted for Method 1, linearity remained high and was similar to Method 3.

### 4.2 Relevance and applicability of the study to other processes

Over the entire cell therapy workflow evaluated in this study, the most suitable method evaluated was Method 3, which produced consistent results with fewer areas of variability. However, for other cell processing workflows there may be other methods that are more or less appropriate for the samples being evaluated. The lack of accuracy for Methods 1 and 2 in relation to bead-bound cells presents challenges in workflows where cells are regularly attached to beads for the purpose of isolation and activation. The staining method in Method 3 uses fluorescent acridine orange and DAPI stains (Robbins and Marcus, 1963; Kubista et al., 1987), which are less impacted by the presence of beads and can identify fluorescent nuclei and non-viable cells. Methods 2 and 3 allow for the user to adjust the gating mechanism used by the instrument to identify live and dead cells, which may be helpful for quantifying new cell types, while Method 1 uses a numerical parameter-based system to set up the analysis algorithm. The utility of a counting method is also impacted by the number of samples that can be processed in a set amount of time, and by the hands-on time required per sample. Methods 1 and 3 allow for a higher throughput of samples to be processed in a shorter amount of time, with less hands-on time required, which is preferable compared to Method 2, which only allows for one sample to be processed at once with greater hands-on time per sample. An example of a selection guide that may be used in determining the most appropriate method of cell counting is given in Table 1. The parameters used were determined to be relevant for this study, however further criteria may be added as needed if utilized in future comparison or establishment studies.

**TABLE 1:** Example selection table that may be used to select an appropriate system for use in cell counting operations. “Performance” referees to the metrics described in ISO 20391-2. Evaluation categories and scoring may be modified to fit into other workflows, values given are only listed in relation to the three systems evaluated in this study and are summarized across cell preparations (i.e., for the different cell preparations investigated, the scoring may vary). In this scaling, the “-” indicates less desirable characteristics, while the “+” indicates more desirable characteristics.

Metric	Method 1	Method 2	Method 3
Scalability	++	-	+
Hands-on time	++	- -	+
Processing time	-	++	+
Overall precision	++	++	+
Counting performance for cells without beads	+	+	+
Counting performance for cells with beads	- -	+	++
Ability to evaluate % viability of cells	--	+	-
Cost per sample	Medium	High	Low

While this study compared 4 different subsets of cells that may be measured in a typical CAR-T production process, there are numerous other applications for this evaluation platform. Non-viral modification has emerged as a way to both generate CAR-T cells (Eyquem et al., 2017) and increase their efficacy (Salas-Mckee et al., 2019; Razeghian et al., 2021). Prior to non-viral modification, cell count and viability are critical values in setting up electroporation parameters with the correct number of cells and ensuring that cells are healthy going into the modification process. The challenge associated with generating CAR-T cells using electroporation is that with larger knock-in constructs, efficiency is lower and cell death increases (Lesueur et al., 2016; Søndergaard et al., 2020). This balance can be monitored through traditional cell counting methods, in addition to more complex tools such as flow cytometry which may be used to measure expression of knocked-in genes. When flow cytometry is performed, researchers use cell count values to prepare and stain cells with multiple antibodies in order to evaluate the efficiency of the genetic modification performed (Litwin et al., 2020). Inaccurate cell counts may negatively impact the staining process and skew results related to phenotyping and evaluation of modification processes. The results of flow cytometry analysis are used to determine purity and identity of the cell therapy product, and are a critical component in quality release testing (Wang and Rivière, 2016).

From beginning to end, cell counts play a vital role in the cell therapy production workflow. This begins with either viral production or cell isolation and culture, and feeds into viral or non-viral modification, and post-modification analytical methods. After modification, cells are expanded and seeding densities must be closely monitored until the point at which doses can be portioned out and delivered to the patient (Wang and Rivière, 2016; Levine et al., 2017). At each control point and at most steps where cells are manipulated, cell counts are used to make decisions that may impact the yield and quality of the final cell therapy product. These counts are also used during process development to determine step and overall process yields and optimize processes to ensure desired outcomes are met. Additionally, regulatory agencies require cell therapy manufacturers to set acceptability criteria related to cell concentration and viability, which are then used for dosing purposes (Guidance for Human Somatic Cell Therapy and Gene Therapy, 1998). For these treatments, getting the dose to the patient is typically time-sensitive and it is crucial that the concentration and viability of the cells being infused is sufficient (Tyagarajan et al., 2020; Bachmeier et al., 2021). All of these applications necessitate accurate measurements, and standards to ensure that cell counting methods being utilized are accurate for designated control points in the workflow.

## 5 Conclusion

The results of this study indicate that the stage of cell processing and physical characteristics of the samples being tested can have significant impacts on the quality and consistency of cell counting measurements. Cell processing applications require cell counts at various stages, which are used to make decisions regarding cell health and seeding, amounts of reagents for viral and non-viral modification, dosing, and analytical evaluation. The cell counting methods evaluated here represent a small fraction of the instruments and methods currently on the market (Cadena-Herrera et al., 2015; Camacho-Fernández et al., 2018; Patel et al., 2022; Stella et al., 2022). As new methods become available, it is critical for researchers to perform proper evaluation of the cell counting measurement process (including instruments, reagents and data analysis procedures) to ensure that sources of variability in the counting method do not lead to errors in the production or use of therapeutic products.

The ISO standard used for this study and the COMET application, can be used to effectively compare cell counting measurement systems and determine the suitability of the systems tested for a specific cell type (Sarkar et al., 2017). The outputs from the analysis are useful in reporting, precision, proportionality, linearity, and more. Added features include evaluation of dilution integrity and variability between users and timepoints as well as evaluation of the consistency of cell viability measurements across dilutions. As a whole, the standard creates clear guidelines for use in laboratories that use either manual or automated cell counting to make decisions during cell processing operations and for dosing.

## Data Availability

The additional contributions presented in the study are included in the article/Supplementary Material, further inquiries can be directed to the corresponding author.

## Author disclaimer

Certain commercial equipment, instruments or materials are identified in this paper to specify the experimental procedure adequately. Such identification is not intended to imply recommendation or endorsement by the National Institute of Standards and Technology, nor is it intended to imply that the materials or equipment identified are necessarily the best available for the purpose. Official contribution of the National Institute of Standards and Technology; not subject to copyright in the United States.
