# Supplementary material for: Assessing the suitability of cell counting methods during different stages of a cell processing workflow using an ISO 20391-2 guided study design and analysis
Source: Front Bioeng Biotechnol. 2023 Aug 3;11:1223227. doi: 10.3389/fbioe.2023.1223227 (PMC10438949; doi:10.3389/fbioe.2023.1223227)
Supplement: Supplementary file 1 [file DataSheet1.pdf]

Supplementary Data

Supplement 1

Linear regression and JMP analysis

Viable cell density (cells/mL) was used to generate linear regressions for each cell type. An initial count was performed using Method 2 to determine the starting concentration for each before creating the dilution series. The viable cell density values for each instrument were plotted against the dilution, and the equation generated was compared to the theoretical equation. These plots are given in **Supplement Figure 1**, with one plot for each cell type evaluated. Based on the dilution series used, the slope of the line drawn between the expected concentrations at each dilution should give a benchmark equation of y = 5x. Data was plotted, and lines of best fit were generated for each cell type and method. The counting method that most closely approximated the benchmark equation for all cell types was Method 3. Methods 1 and 2 were impacted for positively selected T-cells, likely due to the presence of beads and the resulting drop in viability observed for this cell type. Additionally, for Method 2, the presence of beads may lead to some interference with the flow pattern that the cells follow through the microfluidic channels in the counting cassette. For each cell type and dilution, concentration and viability data were analyzed in JMP using a 3-way ANOVA and Tukey HSD. The results of the analysis are shown in **Supplement Table 1** where significance level is indicated by "*" next to the given dilution.

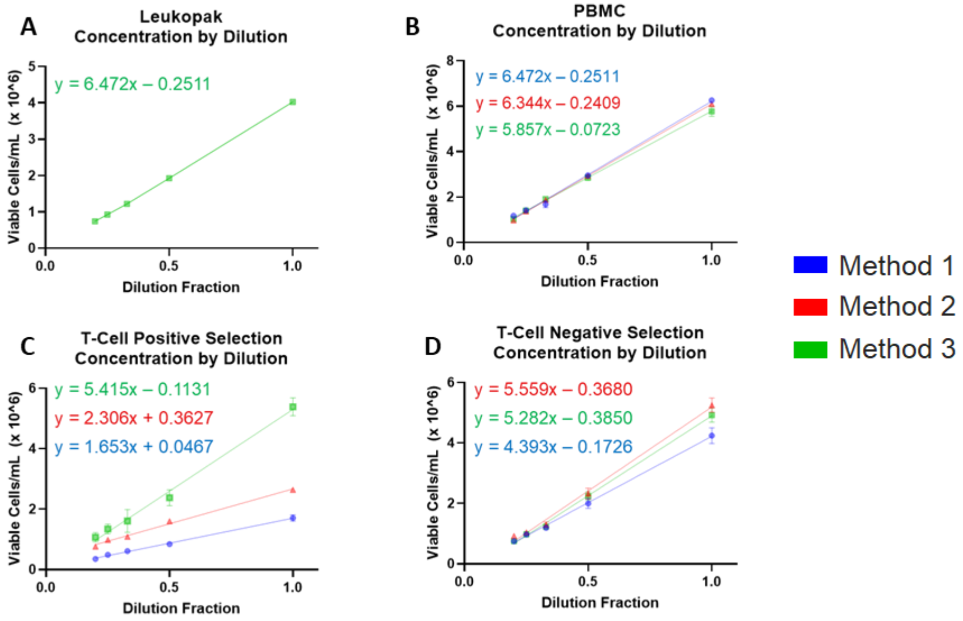

Supplement Figure 1

Plots displaying viable cell density vs. dilution fraction for each cell type, including (A) Leukopak, (B) PBMC, (C) T-Cells with beads attached, and (D) T-Cells without beads attached. Methods are denoted by color of the points and line, as well as the linear regression equation shown to the right for each instrument being evaluated. For Leukopak samples only one instrument was used for analysis.

Supplement Table 1

Comparative analysis of viable cell density and viability for each cell type and dilution factor, separated by pairwise comparison between instruments, where the following are indicated: * p<0.05, ** p<0.005, *** p<0.0005, **** p<0.0001. Analysis performed in JMP using 3-way ANOVA and Tukey HSD.

Viable Cells/mL (by dilution factor, Df)				% Viability (all dilution factors, Df)		
**Sample**	**Method 1 vs. Method 2**	**Method 1 vs. Method 3**	**Method 2 vs. Method 3**	**Method 1 vs. Method 2**	**Method 1 vs. Method 3**	**Method 2 vs. Method 3**
	Df 1.0	Df 1.0	Df 1.0			
	Df 0.5 ****	Df 0.5 **	Df 0.5			
PBMC	Df 0.33	Df 0.33	Df 0.33	All Df ****	All Df ****	All Df
	Df 0.25	Df 0.25	Df 0.25			
	Df 0.2	Df 0.2	Df 0.2			
	Df 1.0 ****	Df 1.0 ****	Df 1.0 ****			
	Df 0.5 ****	Df 0.5 ****	Df 0.5 ****			
T-Cell Positive Selection	Df 0.33 ****	Df 0.33 ***	Df 0.33 ***	All Df *	All Df	All Df
	Df 0.25 ****	Df 0.25 ****	Df 0.25 ****			
	Df 0.2 ****	Df 0.2 ****	Df 0.2 ****			
	Df 1.0 ****	Df 1.0 ****	Df 1.0 *			
	Df 0.5 **	Df 0.5 *	Df 0.5			
T-Cell Negative Selection	Df 0.33 *	Df 0.33	Df 0.33	All Df ****	All Df ****	All Df ****
	Df 0.25	Df 0.25	Df 0.25			
	Df 0.2 ****	Df 0.2 ****	Df 0.2			
